# Knowledge, Attitude and Practice of Research Utilization Among Clinical Nurses and Midwives: Empirical Research Quantitative

**DOI:** 10.1002/nop2.70545

**Published:** 2026-04-11

**Authors:** Lydia Boampong Owusu, Nicholin Scheepers, Atinuke Olusola Adebanji, Immaculate Sabelile Tenza

**Affiliations:** ^1^ Faculty of Health Sciences, School of Nursing Science, North‐West University Potchefstroom South Africa; ^2^ School of Nursing and Midwifery, College of Health Sciences Kwame Nkrumah University of Science and Technology Kumasi Ghana; ^3^ Department of Statistics and Actuarial Science, College of Science Kwame Nkrumah University of Science and Technology Kumasi Ghana; ^4^ Department of Statistics Purdue University Indianapolis USA

**Keywords:** attitude, clinical, cross‐sectional, knowledge, nurses and midwives, practice, research utilization

## Abstract

**Aim:**

To examine knowledge, attitude and practice of research utilization among clinical nurses and midwives using the Rogers theory of Diffusion of innovation.

**Design:**

Cross‐sectional design.

**Methods:**

Quantitative with multistage sampling of 400 clinical nurses and midwives in six hospitals in Kumasi, Ghana. A self‐administered questionnaire was used for data collection. Exploratory and inferential statistical analysis was carried out using Stata 16.

**Results:**

The findings indicated that the majority of respondents had moderate knowledge (71%), a negative attitude (56.2%), and did not practice (76.5%) research utilization. Hierarchical log‐linear analysis revealed a significant positive relationship between research utilization knowledge and practice. Thus, nurses and midwives with more knowledge were more likely to use it. However, knowledge did not significantly affect their attitude towards research utilization. Similarly, attitude did not significantly affect practice. Binary logistic regression analysis revealed gender (male), practising for less than 10 years and profession (nursing) had higher odds of implementing research findings in their practice, with none being statistically significant.

**Conclusion:**

Despite moderate research utilization knowledge, participants had a negative attitude and poor practice aligning with Rogers' theory that attitude influences the decision to adopt an innovation. While research utilization knowledge was significantly associated with improved practice, it did not influence attitude, nor did attitude impact practice. This shows a significant disparity between respondents' knowledge and practice highlighting a gap between knowledge and practice. Therefore, targeted interventions, including continuous professional development and institutional support, are essential to enhance research utilization knowledge, foster positive attitudes, and improve practice. Further research is needed to explore the underlying factors influencing research utilization in nursing and midwifery.

## 1 Introduction

The World Health Organization (WHO) and international health professional bodies such as the International Council of Nurses (ICN) and the International Confederation of Midwives (ICM) emphasize the integration of research findings into clinical nursing and midwifery practice to enhance healthcare quality (ICM 2019; International Council of Nurses 2021; WHO 2020) and achieve the Sustainable Development Goals (SDGs) (Benton et al. 2020).

## 2 Background

Research utilization (RU) is a fundamental component of evidence‐based practice (EBP), ensuring that healthcare professionals incorporate the best available evidence into clinical decision‐making for improved patient outcomes (Chien 2019). Effective RU has been linked to enhanced patient safety, reduced hospitalization rates, cost‐effective care, increased patient satisfaction, and enhanced professionalism of service providers (Chien 2019; Friesen‐Storms et al. 2015; Renolen et al. 2019).

Despite its benefits, RU remains inconsistently practised worldwide (Africa 2023; Hughes et al. 2022; Sun and Larson 2015). Factors influencing its adoption include healthcare providers' knowledge, attitudes, and practices (KAP) regarding RU. Studies in various countries have shown that while some nurses and midwives report implementing research findings, others lack the knowledge, skills, or motivation to do so (Dagne and Tebeje 2021; Kaseka and Mbakaya 2022; Nzengya et al. 2023). Attitude, in particular, plays a crucial role in determining whether research findings are actively integrated into practice (Black et al. 2015).

In Ghana, the Nursing and Midwifery Council (NMC) emphasizes evidence‐informed care (NMC 2022), yet there is limited research specifically examining KAP in RU. Existing studies have largely focused on EBP in general (Agyei et al. 2015; Anaman‐Torgbor et al. 2022; Atakro et al. 2020; Yiridomoh et al. 2020), without assessing nurses' and midwives' knowledge, attitudes, or practice of RU in clinical settings. To achieve EBP in health care, RU must be implemented in nursing and midwifery practice. It is therefore critical to establish the extent of nurses' and midwives' RU with emphasis on their knowledge, attitude, and practice of RU. Understanding these factors is critical for identifying gaps and informing strategies to enhance research‐informed nursing and midwifery care.

## 3 The Study

### 3.1 Aim

This study examined the knowledge, attitude, and practice of RU among clinical nurses and midwives in Kumasi, Ghana.

### 3.2 Objectives

- To assess the level of knowledge of RU among clinical nurses and midwives in Kumasi, Ghana.
- To evaluate the attitude of clinical nurses and midwives towards RU.
- To analyze the practice of RU among clinical nurses and midwives.
- To examine the relationship between knowledge, attitude, and practice of RU in clinical settings.

### 3.3 Primary Objective

- To assess the level of knowledge of RU among clinical nurses and midwives in Kumasi, Ghana.
- To evaluate the attitude of clinical nurses and midwives towards RU.
- To analyse the practice of RU among clinical nurses and midwives.

### 3.4 Secondary Objective

To examine the relationship between knowledge, attitude, and practice of RU.

## 4 Methodology

### 4.1 Design

This study used a cross‐sectional design with a quantitative approach.

### 4.2 Theoretical Framework

This study was guided by Rogers's theory of diffusion of innovation (Rogers 2003). According to Rogers, an idea is accepted after it has gone through the innovation‐decision process (Dearing and Cox 2018). This process starts with knowledge, then persuasion or attitude, and then the individual decides to accept or reject the innovation (Dearing and Cox 2018), which influences the implementation and confirmation of the innovation (Do et al. 2020). The innovation in this study is RU. Diffusion is the process by which an innovation spreads over time among the participants in a social system by means of certain channels (Dearing and Cox 2018). The social system in this study is the healthcare setting. Knowledge was defined as being aware of RU, knowing how to use it, and understanding the principles underlying it, as described by Rogers's diffusion of innovation theory (Dearing and Cox 2018). Attitude is defined as the feelings or thoughts of nurses and midwives towards RU, and it is in the persuasion stage of Rogers's theory where nurses and midwives form a negative or positive attitude about an innovation (Rogers 2003). Practice is defined as the implementation and evaluation of RU in clinical nursing and midwifery practice, and is the implementation stage of Rogers's theory of diffusion of innovation (Dearing and Cox 2018).

Rogers's diffusion of innovation theory has been used in several studies, including studies on e‐health (Putteeraj et al. 2021; Zhang et al. 2015), EBP (Pashaeypoor et al. 2016), pain (Hadorn et al. 2016), and breastfeeding (Oyelana et al. 2021). In a reflection on the diffusion of innovation theory as a theoretical‐methodological framework for research studies on nursing and health, Silva et al. recommended that the theory should be used to assess the results of new knowledge in health and nursing (Silva et al. 2022). In this study, it was used to assess a not‐so‐new knowledge in health and nursing: RU. In terms of RU, a systematic review utilized the theory to assess the barriers to RU in nursing and found that setting‐related factors are the most common barrier to RU in nursing (Jabonete and Roxas 2022). A study in the United States of America used the theory to examine information seeking, RU and the barriers to RU of paediatric nurse educators, and discovered that paediatric nurse educators who consulted nursing journals for information were significantly more likely to use research effectively than other nurse educators, and that nurse characteristics were the most significant barrier to RU (Barta 1995). In Canada, researchers examined the determinants of RU among clinical nurse educators using the theory and found that educators report significantly higher research use than staff nurses and managers (Margaret Milner et al. 2005). However, some of these RU studies that utilized the theory are old, from 2005 (Margaret Milner et al. 2005) and 1995 (Barta 1995), leaving a gap in current RU literature. Also, none has focused on the knowledge, attitudes and practices of RU among nurses and midwives, leaving a gap in the literature.

### 4.3 Instrument With Validity and Reliability

The data was collected with the adapted Evidence‐Based Practice Questionnaire (EBPQ) created by Upton and Upton (2006). With permission from the authors, the questionnaire was modified using Rogers's diffusion of innovations theory to suit the purposes of the study by aligning specific sections of the questionnaire with key stages of the innovation adoption process. These modifications ensured that the questionnaire effectively captured the diffusion process of RU and it was divided into four sections:

- Section A had questions on the demographic information of respondents.
- Section B had questions on the knowledge of respondents which was structured to reflect the different types of knowledge essential for the adoption of an innovation: awareness knowledge, how‐to‐knowledge and principle knowledge.
- Section C sought respondents' attitudes towards RU, which is the persuasion stage of Rogers's theory of diffusion of innovation, capturing respondents' receptiveness to RU.
- Section D assessed the practice of RU, which was aligned with the implementation stage measuring the extent to which RU was adopted in practice.

The questionnaire was developed into Google Forms, with most questions using a five‐point Likert scale (1–5). During analysis, knowledge was assessed using 15 items measured on a five‐point Likert scale ranging from 1 (Very poor) to 5 (Excellent), yielding a total score range of 15 to 75. Knowledge levels were categorized using a modified Bloom's cut‐off (Bloom 1968), where scores of > 75% (≥ 56) were classified as high knowledge, 50%–75% (38–55) as moderate knowledge, and < 50% (< 38) as low knowledge.

Attitude towards RU was measured using 10 items on a five‐point Likert scale (1 = Strongly disagree to 5 = Strongly agree), with total scores ranging from 10 to 50. Scores were re‐categorized into negative (10–29) and positive (≥ 30) attitudes.

RU practice was measured on a five‐point Likert scale (1 = Never to 5 = Always), with total scores ranging from 6 to 30. Practice levels were categorized as low (6–17) and high (18–30).

To ensure the validity of the questionnaire, face validity was established by soliciting input from five expert nurses and midwives, all of whom hold a bachelor's degree and are actively practicing in clinical settings. These experts reviewed the questionnaire to ensure that the questions were relevant, clear, and appropriately aligned with the constructs of the study. Their feedback was instrumental in modifying the tool to better suit the study's focus on research utilization (RU) through the lens of Rogers's Diffusion of Innovations Theory. In addition to face validity, content validity was indirectly enhanced by aligning the modified sections of the questionnaire with key stages of the innovation adoption process outlined in Rogers's theory (2003), specifically focusing on knowledge, attitudes, and practices related to RU. This alignment ensured that the tool accurately captured the diffusion process in the context of RU among paediatric nurses.

To confirm the reliability of the tool, we conducted a pretest using a sample of 40 nurses and midwives (10% of the sample size) from two hospitals located within the study area. The pretest helped assess the clarity, relevance, and internal consistency of the questions, and adjustments were made based on the feedback and input from study supervisors and consultant statisticians. Cronbach's alpha was computed to assess the internal consistency of the modified questionnaire. The results yielded an overall Cronbach's alpha of 0.84, indicating good reliability, as values above 0.7 are considered acceptable for scale‐based instruments.

### 4.4 Sampling and Recruitment

Proportionate sampling was used to allocate a sample size for each facility, based on the number of nurses and midwives in the facility, to arrive at a sample size of 400 for the study.

A systematic sampling method with a sample frame of 9 (which was obtained by dividing the population size by the sample size) was then used to select the sample from each facility.

Nursing and midwifery staff in each hospital were contacted through their nurse managers and trained research assistants who have Bachelor of Science (BSc) degrees in Nursing or Midwifery. Nurses and midwives in the selected health facilities were provided with information on the study and handed the participants' information leaflet which had the contact details of the research assistants. The research assistants went round the wards answering questions of respondents, and those who verbally consented to the study were recruited. Others who were interested in the study contacted the research assistants to express their interest in being recruited into the study. Written consent was obtained, after which the link to the online survey was shared with them individually via WhatsApp or Telegram. Data was collected from December 2022 to February 2023.

### 4.5 Population and Sample

This study was conducted in six hospitals in Kumasi City in the Ashanti region of Ghana, which included one teaching hospital, one regional hospital, one quasi‐hospital, and three district hospitals. These facilities were selected because each represents an agency of health under the Ministry of Health (MoH) of Ghana. Four of the sites are Ghana Health Service facilities, which are the largest health service provision agency in Ghana under the MoH. The quasi‐health facility represents private facilities in the country as well as the other quasi‐government health facilities. The teaching hospital is a tertiary health facility and serves as a referral center for the middle and northern parts of Ghana. All of these hospitals provide general and specialized health services to the public, work with similar health protocols, and their activities are regulated by the MoH.

The target population for this study was nurses and midwives working in the clinical area in the city of Kumasi. As of January 2022, the total population of nurses and midwives for the selected hospitals was 3556.

Taro Yamane's formula was used to calculate sample size (Yamane 1967):

$$n=\frac{N}{1+N\left(e^{2}\right)}$$

*n* = sample size, *E* = level of precision at 95% confidence interval (CI) = (0.05), and *N* = population size = 3556.

$$n=3556/\left(1+\left(3556\times 0.05^{2}\right)\right)=359.5\approx 360,n=360.$$

The sample size had to be adjusted to accommodate for dropout rate using the following formula (Wayne 1975):

$$n1=\frac{n}{1-d},$$

where *n* = 360, the sample size required as per the earlier calculation above; *d* = 0.1, the dropout rate, which was estimated as 10% of the calculated sample size; and *n*1 = adjusted sample size.

$$n1=\frac{360}{1-0.1}=400.\text{Adjusted sample size}=400.$$

A total of 400 nurses and midwives were therefore included in the study.

### 4.6 Inclusion and/or Exclusion Criteria

To be included, the nurses and midwives selected for this study had a minimum educational qualification of a diploma in nursing or midwifery, had worked not less than 2 years post‐qualification, and worked in the clinical area within the city of Kumasi in Ghana.

Nurses and midwives who were on annual leave at the time of data collection or working on a part‐time basis were excluded from the study.

### 4.7 Data Analysis

Data was analysed using Stata version 16. Exploratory data analysis was performed for the socio‐demographic characteristics of the respondents. Descriptive statistics were used to examine the knowledge, attitude and practice of RU. Analysis of variance (ANOVA) was performed between the means of knowledge scores, educational level and age. An independent samples *t*‐test was carried out to identify differences in the knowledge scores of respondents based on the years of practice. A hierarchical log‐linear analysis, which allows for examination of the associations and relationships between categorical variables, was performed. Binary logistic regression was conducted to analyse the relationship between age categories, years of practice, profession and the practice of RU. Statistical significance was established at a *p*‐value of 0.05.

### 4.8 Ethical Considerations

Ethical clearance for the study was obtained and REDACTED. Permission was obtained from the Ashanti regional health directorate and the hospitals. Both verbal and written informed consent were obtained from the respondents before data collection. Identifiers such as names and contact addresses of respondents were not requested to ensure autonomy and confidentiality. Since the data collection was done with a Google Form filled in by respondents in their own time and using their own device, their privacy was ensured. They were informed that participation is optional and that they could opt out of the study at any time. All methods were carried out in accordance with relevant guidelines and regulations.

## 5 Results

The study sought to examine the state of RU of clinical nurses and midwives, focusing on their knowledge, attitude, and practice in Kumasi, Ghana. Data for 400 nurses and midwives was analysed, indicating a 100% response rate of the calculated sample size.

### 5.1 Subjects

Table 1 presents the socio‐demographic characteristics of the respondents. The majority were female (86.5%), aged 30–39 years (63.2%) and had practised for less than 10 years (65.5%). ‘The most common highest qualification was a diploma (50.8%) and the majority of responses were from nurses (55.0%).

**TABLE 1:** Respondents' sociodemographic characteristics, *N* = 400.

Variable	*N* (%)
Age (years)	
Mean (standard deviation)	33.35 (5.4)
Range	20, 50
Age category (years)	
20–29	102 (25.5)
30–39	253 (63.2)
40+	45 (11.3)
Sex	
Male	54 (13.5)
Female	346 (86.5)
Profession	
Nursing	220 (55.0)
Midwifery	180 (45.0)
Highest academic qualification	
Diploma	203 (50.8)
Bachelor's degree	161 (40.3)
Master's degree	16 (4.0)
Specialist	20 (5.1)
Years of practice as a Registered Midwife/General Nurse	
Less than 10 years	262 (65.5)
10 years or more	138 (34.5)
Place of work	
Ghana Health Service facilities	149 (37.2)
Quasi hospital	12 (3.0)
Tertiary health facility	**(59.8)**

### 5.2 RU Knowledge

Table 2 shows the mean scores for knowledge of RU. Respondents demonstrated higher knowledge in using search engines (4.10 ± 0.80) and reviewing their practice (4.02 ± 0.64). Lower knowledge was observed in conducting research studies (3.36 ± 0.83) and analyzing critical research findings (3.43 ± 0.74).

**TABLE 2:** Knowledge of RU among clinical nurses and midwives.

Statements	Mean	Std deviation
Ability to use search engines like Google, Chrome, Microsoft Edge, Firefox to search for articles or information	4.10	0.80
Awareness of major sites to search for information on nursing and midwifery, like Google Scholar, Research Gate, Semantic Scholar, Microsoft Academic, Pubmed, Medscape, Medline Plus, HINARI, Cochrane	3.64	0.88
Ability to conduct a research study	3.36	0.83
Ability to review or reflect on your own practice (to look back on the way you used to practice in the past)	4.02	0.64
Identify gaps/lapses in professional practice or areas in professional practice that need a revision or improvement	3.83	0.64
Converting identified gaps/lapses in practice into problems that require research findings to solve	3.47	0.75
Knowledge of how to retrieve research findings/evidence that is useful for your practice	3.51	0.75
Ability to analyse critical research findings/evidence against set standards/standard procedures	3.43	0.74
Ability to determine how valid (close to the truth) evidence/research finding is	3.51	0.73
Ability to determine how useful (clinically applicable) the evidence/research finding is	3.67	0.72
Ability to apply the identified research findings/evidence to individual patient cases	3.78	0.69
Sharing of research ideas and research findings with colleagues	3.80	0.78
Monitoring and reviewing of practice skills after implementing research findings/evidence	3.59	0.72
Dissemination of new ideas (gained from research findings) about care to colleagues	3.80	0.72
How well did your nursing/midwifery training/school prepare you to implement research findings/evidence in practice?	3.68	0.91

### 5.3 Attitude Towards RU

As shown in Table 3, respondents strongly agreed that utilizing research is fundamental to professional practice (4.28 ± 0.79) and welcomed questions on their practice (4.09 ± 0.74).

**TABLE 3:** Attitude among clinical nurses and midwives on RU.

Statement	Mean	Std deviation
Providing patient care based on research findings disorganizes my daily work	2.37	1.02
My workload is too much for me to keep up to date with all the new evidence	2.55	1.09
New research evidence is so important that I make time in work to access it	3.59	0.82
I resent (dislike) having my clinical practice questioned	2.29	1.01
I welcome questions on my practice (I am open to have my patient care practices questioned)	4.09	0.74
Utilizing research is a waste of time	1.68	0.82
Utilizing research is fundamental to professional practice (utilizing research findings is important in nursing/midwifery practice)	4.28	0.79
I stick to tried and trusted methods rather than changing to anything new	3.00	1.19
I do not like changing the way I practice; I like doing things the way I have been doing it	1.92	0.86
I have changed the way I practice nursing/midwifery care due to research influence	3.87	0.89

### 5.4 RU Practice

Table 4 shows item statistics related to the use of research evidence in clinical practice (practices). Sharing of information with colleagues (3.77 ± 0.96) is the RU activity that respondents mostly engaged in, while critically analyzing literature is the least often used (3.11 ± 0.93).

**TABLE 4:** Item analysis for RU practices.

Statement	Mean	Std deviation
Searching for relevant research evidence once you identified a clinical problem	3.35	0.85
Identifying relevant research evidence to resolve a clinical problem	3.32	0.90
Critically analysing literature you have discovered to resolve the clinical problem	3.11	0.93
Implement the research/findings/evidence you have found in your practice	3.52	0.94
Evaluating the outcomes of your practice after implementing the identified research evidence	3.46	0.96
Sharing of this information (identified research evidence) with colleagues	3.77	0.96

### 5.5 Knowledge, Attitude and Practice Analysis

Table 5 summarizes the overall levels of knowledge, attitude, and practice of RU. The majority of respondents had moderate knowledge (71%), a negative attitude (56.2%), and did not practice RU (76.5%).

**TABLE 5:** Knowledge, attitude and practice of RU.

Variable	*n* (%)
Knowledge	
High	110 (27.5)
Moderate	284 (71.0)
Low	6 (1.5)
Attitude	
Positive	175 (43.8)
Negative	225 (56.2)
Practice	
Yes	94 (23.5)
No	306 (76.5)

### 5.6 Relationship Between Knowledge and Socio‐Demographic Characteristics

ANOVA for knowledge score and the categories of educational level and age.

Analysis of variance (ANOVA) between the means of educational level and knowledge scores indicates an *F*‐value of 2.288, which suggests a significant difference between the means of at least two groups, further supported by the *p*‐value of 0.045. Therefore, it can be concluded that educational level significantly affects knowledge score. ANOVA between knowledge scores and age categories shows no significant difference, with an *F*‐value of 0.007 and a *p*‐value of 0.994.

### 5.7 Years of Practice and Knowledge Scores

The respondents were grouped based on years of practice (< 10 years and ≥ 10 years), with mean knowledge scores of 55.02 and 55.51, respectively. With means so similar, performing a two‐sample test was not considered necessary; hence, it was concluded that the number of years in practice was not associated with research utilization (RU) knowledge scores.

### 5.8 Relationship Between Knowledge, Attitude, and Practice

#### 5.8.1 Log‐Linear Analysis

A hierarchical log‐linear analysis (HILOGLINEAR) was conducted to examine the associations between knowledge, attitude, and practice of RU among clinical nurses and midwives. Model fit was assessed using the Likelihood Ratio and Pearson chi‐square statistics. Both tests were non‐significant (Likelihood Ratio *χ* ^2^ = 0.211, *p* = 0.646; Pearson *χ* ^2^ = 0.210, *p* = 0.647), indicating no significant difference between the observed and expected frequencies and suggesting that the model provided a good fit to the data.

Analysis of K‐way and higher‐order effects showed no significant three‐way interaction between knowledge, attitude, and practice (*p* = 0.646), indicating that the combined interaction of all three variables was not statistically significant (Table 6). However, significant two‐way interactions were observed between knowledge, attitude, and practice (*p* < 0.001).

**TABLE 6:** Log‐linear analysis of knowledge, attitude, and practice.

	*K*	Df	Likelihood ratio		Pearson		No. of iterations
			Chi‐square	Sig.	Chi‐square	Sig.	
K‐Way and Higher‐Order Effects^a^	1	7	127.863	0.000	132.400	0.000	0
	2	4	47.637	0.000	47.411	0.000	2
	3	1	0.211	0.646	0.210	0.647	3
K‐Way Effects^b^	1	3	80.226	0.000	84.989	0.000	0
	2	3	47.426	0.000	47.201	0.000	0
	3	1	0.211	0.646	0.210	0.647	0

^a^ Tests that K‐Way and Higher‐Order Effects are zero.

^b^ Tests that K‐Way Effects are zero.

### 5.9 Partial Associations

Partial associations (Table 7) showed a significant relationship between knowledge and practice (*p* < 0.001), but not between knowledge and attitude (*p* = 0.697) or attitude and practice (*p* = 0.410).

**TABLE 7:** Partial associations of knowledge, attitude, and practice.

Effect	Df	Partial Chi‐square	Sig.	No. of iterations
Knowledge*Attitude	1	0.152	0.697	2
Knowledge*Practice	1	45.892	0.000	2
Attitude*Practice	1	0.680	0.410	2
Knowledge	1	6.779	0.009	2
Attitude	1	20.424	0.000	2
Practice	1	53.022	0.000	2

### 5.10 Baseline Category (Reference) of the Response Variable

Any category of the response variable which consists of ‘Practice’ and ‘Not practice’ can be chosen to be the baseline or reference category; the model will fit equally well, achieving the same likelihood and producing the same fitted values, just the values and interpretation of the parameters will change (Lanza et al. 2007).

### 5.11 Binary Logistic Regression

Binary logistic regression (Table 8) found no statistically significant relationship between demographic factors (age, years of practice, profession) and the practice of RU (*p* > 0.05).

**TABLE 8:** Binary logistic regression between RU practice and demographics.

Predictors	Sig.	Odds ratio	95% CI for exp (*B*)	
			Lower	Upper
Gender (male)	0.613	1.196	0.598	2.391
Age category	0.072			
Age category (20–29 years)	0.431	0.706	0.297	1.679
Age category (30–39 years)	0.482	1.290	0.635	2.621
Years registered (10 years or less)	0.063	1.658	0.972	2.827
Profession (nursing)	0.892	1.032	0.652	1.634
Constant	0.335	1.387		

## 6 Discussion

The study sought to examine the state of clinical nurses and midwives RU with emphasis on their knowledge, attitudes, and practices of RU.

The majority of respondents had a diploma as their highest academic qualification, which is the basic educational level of nurses and midwives in most parts of Africa (Nzengya et al. 2023). This could negatively affect their in‐depth knowledge of research activities, including RU as found in South Africa, where degree nurses are more involved in scholarly activities and implement best practice guidelines than diploma nurses (Grobler et al. 2016). Again, in the curriculum of the diploma in nursing and midwifery in Ghana, research is studied in one semester, whereas at the Bachelor's degree level, research is studied in two semesters. This provides the Bachelor's prepared nurse or midwife with more research knowledge and skills, which can positively influence their RU. Evidence supports this distinction: nurses educated at the bachelor's level are associated with positive patient outcomes (Kamanyire and Achora 2015), and a systematic review of 21 studies found that bachelor‐prepared nurses have been demonstrate higher competency levels compared to diploma‐trained nurses (Rizany et al. 2018). The fact that the majority in this study had a diploma indicates that the basic qualification of nurses and midwives is of lower educational status and explains the low RU practice found in this study. This is contrary to what was found in Florida, where only 21.36% of a sample of 337 nurses had a diploma (Cline et al. 2017); more than 70% of nurses in the entire United States of America have a Bachelor's degree or higher (Smiley et al. 2023). The low educational level found in this study was also found in another study in Ghana, where nurses attributed their restricted knowledge on RU to limited knowledge of research (Yiridomoh et al. 2020). The low educational level in nursing and midwifery could affect their translation of research into practice (Aljezawi et al. 2019; Dagne and Ayalew 2020), as respondents had lower mean scores in their knowledge of conducting research and analysing critical research findings. A systematic review also identified education as key in enhancing nurses' RU (Tuppal et al. 2019). These findings support the calls for higher education in nursing and midwifery, and for the Bachelor's degree to be the basic training level for nurses and midwives in order to promote RU (Acheampong et al. 2021; American Association of Colleges of Nursing 2019; Huang et al. 2017; Karlberg‐Traav 2020; Kaseka and Mbakaya 2022). This will enhance RU practice, as found in India (Kalliguddi et al. 2019), Malawi (Kaseka and Mbakaya 2022) and Ethiopia (Dagne and Tebeje 2021), where knowledge influenced practice. According to the American Association of Colleges of Nursing, higher education makes a difference in nursing practice (Rosseter 2014).

The moderate knowledge found in this study could be attributed to the majority of participants having a diploma in nursing or midwifery. This could be linked to their knowledge of some RU activities, like the use of search engines to search for articles and sharing identified research findings with colleagues. This shows higher knowledge in access and dissemination, which are the first and third steps of RU respectively. This moderate knowledge could affect the nursing and midwifery practice within the NMC of Ghana's scope of practice (NMC 2022) and the ICN code of ethics (International Council of Nurses 2021), which require nurses and midwives to implement research findings in their practice. However, again the moderate knowledge is manifested by their inadequate knowledge in the second and last steps of RU, which are analysis and implementation. This is comparable to previous studies, where nurses identified difficulty in critically analysing research findings to promote EBP (Cline et al. 2017; Stavor et al. 2017). This will make it difficult for nurses and midwives to achieve a research culture focusing on incorporating RU into daily clinical practice to support EBP (Berthelsen and Hølge‐Hazelton 2017, 2021; Cline et al. 2017). Nursing and midwifery bodies like the NMC of Ghana, the Ghana College of Nurses and Midwives, and the Ghana Registered Nurses and Midwives Association need to organize continuous professional development courses on RU for nurses and midwives to promote its practice, as recommended by other studies (Cline et al. 2017; Farokhzadian et al. 2021; Mudderman et al. 2020; Shepherd et al. 2022). A nursing and midwifery society that focuses on research, like the Southern Nursing Research Society, can also be instituted to further promote and enhance RU through dissemination and mentorship (Garey 2022).

Attitude, according to Rogers theory of diffusion of innovation, is the persuasion stage that can influence or determine acceptance or rejection of an innovation (Dearing and Cox 2018). A positive attitude towards RU can foster a supportive environment for its integration into clinical practice, influence the willingness to change and adopt new practices based on research findings (Pascal Iloh et al. 2020), and promote commitment to continuous learning and professional development (Al‐Maskari and Patterson 2018). The negative attitude identified in this study could mean that nurses and midwives will not practice RU. This finding contrasts with studies in other contexts. For example, in Norway, both experienced nurses (Moe and Enmarker 2020) and newly graduated nurses (Wangensteen et al. 2011) had a positive attitude towards RU. Similar results were reported in Egypt (Mohamed Thabet and Abd El‐Naser Al 2016) and Spain (Moreno‐Casbas et al. 2011), where majority of nurses had a positive attitude towards RU Healthcare professionals with a positive attitude towards RU recognize the importance of research in informing clinical decision making (Black et al. 2015). The study also found that RU knowledge and attitude influence its practice, as stated by Rogers that an individual decides to accept and implement an innovation after having knowledge and a positive attitude towards it (Dearing and Cox 2018). In our study, this could mean that moderate knowledge, coupled with a negative attitude, have resulted in poor practice. This is consistent with findings in Ethiopia that knowledge of RU and a negative attitude serve as barriers to RU (Dagne and Tebeje 2021), and also with a systematic review that identified nurses' lack of knowledge influences their RU (Lode et al. 2015). Further studies on the factors influencing attitude towards RU are recommended.

Compared to the low rate of RU practice observed in this study, which contrasts with the 80% adoption rate reported in Kenya (Nzengya et al. 2023), our findings align more closely with those from Egypt, where 60% of individuals do not implement RU (Mohsen et al. 2016). The findings also contradict the assertion that an increase in nursing research publications in Africa will certainly result in increased RU practice and ultimately enhanced EBP (Berthelsen and Hølge‐Hazelton 2021). In terms of practice and the RU steps, there was low access, analysis and implementation with higher dissemination. This means that nurses and midwives mostly disseminate research findings but have poor practice of the other RU steps. Dissemination, known as diffusion in Rogers theory, has been found to be an important step in RU and it enhances practice change (Shepherd et al. 2022). This is confirmed by the diffusion of innovation theory that states that an individual who hears of an innovation communicates it to other members of the social system, which can enhance the decision to implement. In this case, RU knowledge is shared with colleagues in the health facility. Further strategies to enhance dissemination should be adopted to promote RU. In addition, using the RU steps to implement research findings in clinical care is vital; in Australia, RU in healthcare resulted in best performance (McKeon 2013). This study's findings of low RU practice with moderate knowledge is similar to those of a study in Kuala Lumpar, Malaysia (Bashar 2019). The low practice could be attributed to the theory‐practice gaps that have been identified in several countries, including Australia (Robinson et al. 2020) and Ethiopia (Dagne and Tebeje 2021), and it is recommended that concerted efforts should be made among stakeholders and health professionals to build capacity to promote translation of RU knowledge into practice (Robinson et al. 2020). In addition, the low RU practice could be the basis for the development of a conceptual framework to guide the practice of RU.

This study found no significant association between years of practice and RU knowledge in nursing and midwifery. This means that clinical nursing or midwifery experience does not automatically translate into an increase in RU knowledge, since years of practice without the use of research in practice could not be expected to increase research knowledge. While work experience contributes to clinical expertise, RU requires specific competencies that may not be acquired solely through years of practice (Alavi et al. 2022; Manoochehri et al. 2015; Wangensteen et al. 2012). Effective RU implementation requires research literacy and understanding of methodologies (Kaseka and Mbakaya 2022), which can be acquired during nursing education and can vary, depending on how they are taught (Coyne et al. 2016). Also, the work environment plays a crucial role in facilitating RU (Da'seh and Rababa 2021; Dagne and Tebeje 2021). Healthcare organizations that prioritize EBP and provide resources for research education, mentorship, and access to relevant literature are more likely to have nurses and midwives with enhanced research knowledge (Nzengya et al. 2023). Further studies should explore leadership support for RU in health facilities (Owusu et al. 2023).

### 6.1 Strengths and Limitations of the Work

The study was conducted in six hospitals in the city of Kumasi, located in one of the 16 regions in Ghana. However, lessons from this study could be useful for other regions with similar contexts. The study did not include nurses and midwives working in rural areas. That notwithstanding, the study was conducted in six hospitals across Kumasi comprising teaching, regional, and district hospitals providing a comprehensive view of nurses and midwives' RU from different levels of health care.

To our knowledge, this is the first study in Sub‐Saharan Africa using Rogers' theory of diffusion of innovation to assess the knowledge, attitude, and practice of RU by nurses and midwives. The study also used a large sample size (400) and had a 100% response rate and included nurses working in diverse health facilities representing most of the agencies of health under the MoH in Ghana. This increases the generalizability of the study findings. Again, the study addresses a significant gap in the literature on RU in LMICS, providing valuable context‐specific insights that have global relevance.

### 6.2 Recommendations for Further Research

Further research on the source of information guiding clinical nursing and midwifery practice, to explore the underlying factors contributing to the disconnect between knowledge and practice and influencing RU in nursing and midwifery, is recommended. Factors influencing RU and exploring leadership support for RU in health facilities are recommended. Longitudinal studies could track changes in RU knowledge and practices over time to evaluate the effectiveness of interventions.

## 7 Implications for Policy and Practice

The use of Rogers' theory together with the steps in RU enabled the identification of areas that require improvement in empowering nurses and midwives RU. For example, according to Rogers, an individual develops an attitude towards an innovation after knowing about it. With the moderate knowledge found in this study, one would have expected a positive attitude, in contrast to the negative attitude identified. Regarding knowledge of the professionals in terms of the steps of RU, there was higher knowledge for accessing and disseminating research articles, with low knowledge in analysis and implementation. In terms of practice and the RU steps, there was low practice of access, analysis, and implementation, with higher dissemination. Dissemination, identified by Rogers as diffusion, greatly impacts RU practice, and health managers would have to leverage it to enhance RU. Continuous professional development courses on RU for nurses and midwives to promote its practice are needed. A nursing and midwifery society that focuses on research, like the Southern Nursing Research Society, can be instituted to further promote RU through dissemination and mentorship. Policy for Bachelor's degree to be the basic level of nursing and midwifery education needs to be formulated.

## 8 Conclusion

There is a significant disparity between the respondents' knowledge and their actual utilization of research. This needs to be addressed to enhance RU and ultimately promote EBP in clinical care. The negative attitude and poor RU practice found confirm Rogers' theory, where an individual at the persuasion stage forms an attitude which influences the decision to implement an innovation. In this study, negative attitudes affected the decision to implement RU, leading to poor practice.

## Author Contributions

L.B.O.: conceptualization; writing – original draft; methodology; formal analysis; writing – review and editing. N.S.: conceptualization; methodology; writing – review and editing; supervision. A.O.A.: conceptualization; methodology; formal analysis; writing‐review and editing; provided statistical consultations for the study. I.S.T.: conceptualization; methodology; writing – review and editing; supervision.

## Funding

The authors have nothing to report.

## Disclosure

Statistics: There is a statistician on the author team. She is Atinuke Olusola Adebanji, a statistics Professor.

## Ethics Statement

The data utilized in the submitted manuscript have been lawfully acquired in accordance with The Nagoya Protocol on Access to Genetic Resources and the Fair and Equitable Sharing of Benefits Arising from Their Utilization to the Convention on Biological Diversity. Permission was obtained from the Ashanti regional health directorate and the management of the various health facilities involved in the study, Health Research and Ethics Committee (HREC) of the North‐West University (NWU) (NWU‐00070‐22‐A1), the Ghana Health Service Ethics Review Committee (GHS‐ERC 003/07/22); the Komfo Anokye Teaching Hospital Institutional Review Board in Kumasi (KATH IRB/AP/152/22), and the Committee on Human Research, Publication and Ethics of the School of Medicine and Dentistry at the Kwame Nkrumah University of Science and Technology in Kumasi, Ghana (CHRPE/AP/762/22).

## Conflicts of Interest

The authors declare no conflicts of interest.

## Data Availability

The data that support the findings of this study are available from the corresponding author upon reasonable request.
